# Prolidase Deficiency Presenting With Pulmonary Arteriovenous Malformations and Seizures: Report of Two Cases From Iran

**DOI:** 10.1155/crpe/9929135

**Published:** 2025-10-02

**Authors:** Ali Pajouhi, Rouhollah Rohani, Vahid Ziaee, Mohammad Shahrooei, Zeinab Paymani, Bahar Amiri, Mohammadreza Modaresi

**Affiliations:** ^1^Student Research Committee, Lorestan University of Medical Sciences, Khorramabad, Iran; ^2^USERN Office, Lorestan University of Medical Sciences, Khorramabad, Iran; ^3^Pediatric Respiratory and Sleep Medicine Research Center, Children's Medical Center, Pediatric Center of Excellence, Tehran University of Medical Sciences, Tehran, Iran; ^4^Children's Medical Center, Pediatric Center of Excellence, Tehran University of Medical Sciences, Tehran, Iran; ^5^Department of Pediatrics, Tehran University of Medical Sciences, Tehran, Iran; ^6^Pediatric Rheumatology Society of Iran, Tehran, Iran; ^7^Pediatric Rheumatology Research Group, Rheumatology Research Center, Tehran University of Medical Sciences, Tehran, Iran; ^8^Clinical and Diagnostic Immunology, Department of Microbiology, Immunology, and Transplantation, KU Leuven, Leuven 3000, Belgium; ^9^Dr. Shahrooei Lab, 22 Bahman St. 34, Ashrafi Esfahani Blvd, Tehran, Iran; ^10^Department of Nuclear Medicine, Children's Medical Center, Pediatric Center of Excellence, Tehran University of Medical Sciences, Tehran, Iran

**Keywords:** AVMs, genetic disorders, Iran, prolidase deficiency, pulmonary arteriovenous malformations, rare diseases, seizure

## Abstract

Prolidase deficiency (PD) is a rare autosomal recessive metabolic disorder caused by pathogenic variants in the *PEPD* gene, which is responsible for making prolidase and collagen resynthesize. It leads to a wide range of clinical symptoms, including skin ulcers, intellectual disability, and recurrent infections. Diagnostics are based on the presence of dipeptides in the urine and prolidase activity in various cells, as well as the detection of the pathogenic variant in the *PEPD* gene; however, no standard method is currently known for its diagnosis nor for its treatment. Case 1 presents a 17-year-old male with dyspnea, cyanosis, and pulmonary arteriovenous malformations (AVMs), an atypical presentation for PD. Case 2, a one-year-old female with fever, seizures, productive coughs, erythematous ulcers, and developmental delays, highlights the disease's broad symptoms. This study widens our understanding of PD, especially for lesser-known populations, such as the population in Iran. It indicates considering PD in the differential diagnosis of various diseases and also a global collaboration for managing PD. The study also recommends a standard approach to diagnosing PD and a personal management system due to its broad manifestations. In addition, it indicates the need to consider different ethnic and geographical groups in future PD studies to further enhance our understanding of this rare disease.

## 1. Background

Prolidase deficiency (PD) is an extremely rare autosomal recessive disease caused by pathogenic variants in the *PEPD* gene, which is responsible for making prolidase, a crucial enzyme in proline metabolism that is essential in the breakdown of collagens [1]. This makes PD cause significant clinical symptoms, including skin ulcers, intellectual disability, skeletal deformities, hematological disorders, splenomegaly, and recurrent infections, which can be life-threatening at some points [2, 3]. The presence of dipeptides in the urine (imidodipeptiduria) and also decreased or absent prolidase activity in erythrocytes, leukocytes, and cultured fibroblasts are the main factors in diagnosing PD [3].

As prolidase is crucial for collagen metabolism, PD is significantly linked to skin disorders, such as skin ulcers [4], or other parts where proline metabolism plays a leading role, as mentioned earlier. However, the exact mechanism that leads to various diseases caused by PD remains unknown [5]. Systemic lupus erythematosus (SLE), for instance, has been shown to be related to PD [6].

To date, PD has no definite treatment [7]. As approximately 160 cases were reported for this disease, the academic knowledge of PD is limited in the literature [8]. In this article, we report two cases of PD, making a novel contribution to the medical literature. To our knowledge, this is the first report to fully document PD in the population in Iran, highlighting its rarity.

## 2. Case 1

A 17-year-old male patient born to consanguineous parents with dyspnea over the last five years, which has progressed with exercise intolerance and central cyanosis to the point that even walking for a few steps worsens symptoms, presented to our pediatric pulmonary clinic. The dyspnea was seen to exacerbate in the standing position. The patient was also complaining about multiple skin lesions and digit changes.

In his past medical history (PMH), he was diagnosed with hepatomegaly due to potential tyrosinemia, but no treatment was received. The patient had no follow-up for years. He had a neck abscess a few years earlier, which was drained. No family history of chronic illnesses was reported.

On physical examination (P/E), he was fully oriented and had stable vital signs, with a marfanoid appearance. Hypertelorism, a broad nasal bridge, central cyanosis on the lips, multiple skin lesions in various body regions, hyperkeratotic areas, clubbing of the fingers, suprasternal retraction, and cyanosis of the extremities were also reported. Further examination revealed a mild systolic murmur with normal lung sounds, hepatomegaly 2-3 cm below the costal margin, and massive splenomegaly.

Thrombocytopenia was reported in his laboratory results. His arterial blood gas (ABG) showed hypoxemia with an O_2_ saturation of 80%. His liver function test (LFT) showed an increased prothrombin time (PT) of 22 s, an international normalized ratio (INR) of 1.68, and an increased aspartate aminotransferase (AST) of 97 units/L. Increased IgG levels of 2260 mg/dL, IgA levels of 454 mg/dL, and IgE levels of 450 IU/mL were reported in his immunological tests (Table 1).

Left ventricular hypertrophy was reported in his echocardiography, with normal pulmonary pressure and systolic function. Also, a large atrial septal defect (ASD) (size: 6 mm) was reported. No aortic root dilatation or lens dislocation was reported, ruling out Marfan syndrome.

Lung computed tomography (CT) scan revealed interstitial edema and lymphangiectasis, accompanied by bilateral interlobular septal thickening, a nonspecific finding.

The hypoxemia approach was initiated due to the patient's low paO_2_. The alveolar-arterial (A-a) gradient was significantly increased, suggesting a right-to-left shunt. Agitated saline contrast echocardiography confirmed the shunt at the pulmonary vascular bed.

A pulmonary perfusion scan showed a widespread escape of macroaggregated albumin from the pulmonary vasculature, indicating pulmonary arteriovenous malformations (AVMs). Sonographic findings are summarized in Table 2. Selective pulmonary angiography was done for the patient, which confirmed the presence of AVMs. Lung transplantation was recommended as embolization was unfeasible.

Further investigations using Whole Exome Sequencing (WES) revealed a variation in the *PEPD* gene, confirming PD, which correlates with the patient's signs and symptoms.

WES Test:

Homozygous genotype in the *PEPD* gene (NM_000285:exon15: c.1359_1361del).

## 3. Case 2

A one-year-old female patient born to consanguineous parents presented to the pediatric clinic with fever and seizures for the last 6 months, associated with productive coughs. She had unstable vital signs with a pulse rate of 120 beats per minute (BPM) [100-180 BPM] [9], blood pressure of 85/P mmHg [72–104 mmHg] [9], respiratory rate (RR) of 30/minute [30–53/minute] [9], and temperature of 38.5°C [36.5°C to 37.5°C] [10]. The symptoms were accompanied by multiple erythematous ulcers on the anal verge, oral mucositis, and crusted erythematous lesions on lips that first appeared at the age of 9 months.

At the age of 7 months, she was investigated for an enlarged abdomen, which turned out to be hepatosplenomegaly. Her weight and height were both below the normal centile for her age, which raised concerns for growth disorders.

Her P/E revealed dermatological manifestations, including dry, crusted lesions on the anal verge, mouth, and lips. Abdominal examination revealed hepatomegaly 6 cm below the costal margin. There were no signs of lymphadenopathy, photosensitivity, or facial dysmorphisms.

Initial laboratory investigations (Table 1) showed leukopenia, thrombocytopenia, and anemia with a hemoglobin level of 8.3 g/dL. ABG analysis revealed hypoxemia with a pH of 7.4. LFTs showed increased PT and INR. Immunological tests revealed increased levels of IgG, IgA, and IgE. Anti-liver-kidney microsomal (LKM) and alpha-1 antitrypsin antibodies were positive.

Her blood culture was positive for *Enterococcus faecium*. Bone marrow aspiration and biopsy showed diluted bone marrow, erythroid hyperplasia, and hemophagocytic cells. Abdominal sonography revealed an enlarged liver and spleen, suggestive of hepatosplenomegaly (Table 2).

Based on her clinical symptoms, she was investigated for PD; the WES test showed a homozygous variant in the *PEPD* gene, confirming the diagnosis.

WES Test:

Homozygous genotype in the *PEPD* gene (NM_001166057: exon7: c.G442C).

## 4. Discussion

PD, a rare autosomal recessive disorder, has an estimated incidence of about one to two cases per million births [6]. This disease is mainly known for impairing wound healing abilities, an effect caused by pathogenic variants in the *PEPD* gene, which encodes prolidase (peptidase D), an important enzyme for collagen turnover due to its capability to cleave imidodipeptides with C-terminal proline or hydroxyproline [5, 11]. It acts as an enzyme to recycle proline from imidodipeptides, mainly from collagen products, to resynthesize collagen and other proteins rich in proline [12]. Prolidase is also crucial in maintaining connective tissue integrity, making its deficiency cause a handful of pathological mechanisms [13]. The *PEPD* gene is located on chromosome 19q13.11 and spans roughly 130 kilobases (kb). It contains three transcript variants, which lead to three protein isoforms. Isoform 1 is the most studied of all and is also the longest [5].

Prolidase function is related to cell growth activities such as wound healing, inflammation, angiogenesis, cell proliferation, and even carcinogenesis [5]. Pathways such as the insulin-like growth factor 1 receptor (IGF-1R), β1 integrin receptor, and downstream proteins, including focal adhesion kinase (FAK), growth factor receptor-bound protein 2 (Grb2), and extracellular signal-regulated kinases 1 and 2 (ERK1/2), are responsible for its regulation, which is essential for cell growth and proliferation [5, 14, 15].

Pathogenic variants in the *PEPD* gene lead to impaired protein turnover, particularly of collagen, which is rich in proline. PD was first introduced by Goodman et al. in 1968, presenting a man with a multisystem disorder in the skeleton, eyes, ears, and cardiovascular system, which was suggested to be a lathyrism-like syndrome at that time [16]. Recent case reports have presented PD cases worldwide. Basu et.al. presented a 12-year-old Indian boy with a novel variation in the *PEPD* gene with symptoms including facial dysmorphism, corneal opacity, and nonhealing leg ulcers [17]. A Turkish case report documented a 16-year-old girl diagnosed with PD presenting pulmonary hemosiderosis and cytoplasmic antineutrophil cytoplasmic antibody (c-ANCA) positivity, which was also accompanied by Friedreich's ataxia [18]. Azhar et al. reported PD in three siblings aged 17–20 with splenomegaly and hypoalbuminemia [19]. A 2025 report presented four new PD cases with a wide range of symptoms, including immunological, gastrointestinal, neurological, and hematological [8]. Another paper published in the same year introduces a nearly 3-year-old PD patient with interstitial lung disease (ILD), an uncommon presentation of the disease [20].

The age of diagnosing PD varies from birth to 22 years of age, with some cases remaining asymptomatic [6]. It has a wide range of clinical presentations, including dysmorphic features, intellectual disabilities, recurrent infections, persistent skin ulcers, autoimmune phenomena, and splenomegaly [21–23]. Its broad and often nonspecific symptoms make it challenging to diagnose. The average age of diagnosing PD is approximately 11.6 years, with half of the cases requiring more than 8.5 years to confirm the disease [24]. The lack of unified criteria for diagnosing PD adds to its complexity in diagnosis [21].

This study aligns with previous PD studies and provides new insights into this disease. Our first case presented with progressive dyspnea, central cyanosis, extensive AVMs, and a history of hepatomegaly (Table 2). These findings, especially the AVMs, have been rarely reported in PD cases [25], highlighting its uniqueness for this disease in this demographic.

In addition, the second case presented with persistent fever, seizures, developmental delays, hepatosplenomegaly, and immunological anomalies (Table 2) helps us better understand PD, as the seizure episodes are a rare presentation of the disease [6], adding more knowledge to its clinical presentations.

The variant in the first patient (NM_000285:exon15: c.1359_1361del) causes the deletion of glutamic acid at position 453(p.Glu453del). It was previously reported in patients with PD in 1994 [26] and was further experimented on in 1996, showing no enzyme activity in transfected cells [27]. It was classified in a ClinVar submission in 1996 as pathogenic. However, more data are needed to fully support its pathogenicity [28].

The variant in the second patient (NM_001166057: exon7: c.G442C) is a missense, single-nucleotide variant that leads to an amino acid change from alanine to proline at position 148(p.Ala148Pro). It has been detected in patients with PD [29], and the most recent ClinVar submission in 2024 classifies it as pathogenic [30].

The demographic in which this report is being presented is valuable as it is the first article to fully document PD in the population in Iran; the only paper we could find regarding PD in Iran was a 1995 abstract-only report, which was only available in Persian [31]. Although this report aligns with the global cases previously reported, its occurrence in the population in Iran helps us further identify the disease's clinical manifestations in different populations and better understand its diversity, both geographically and ethnically. The occurrence of PD in such populations highlights the importance of considering PD in the differential diagnosis (DDx) of the diseases manifested similarly and also in genetic counseling. Including underrepresented populations in future genetic studies is also important to provide a broader understanding of rare genetic disorders, such as PD.

Lacking a standard approach to diagnosing and managing PD challenges us to provide adequate healthcare [22]. This study indicates tailored strategies for patient management, including symptomatic treatment and preventive measures. We will understand the importance of this kind of approach when we acknowledge how wide and complex PD manifests, such as the AVMs in one patient, which eventually led to lung transplantation suggestion.

This article also emphasizes the importance of global collaboration in diagnosing and managing PD [32], which is especially beneficial in countries where PD is less frequently reported. Future studies should focus more on genetic patterns in these countries to gain a more comprehensive understanding of the diversity of PD across different ethnic groups.

## 5. Conclusions

In conclusion, this study contributes to the global understanding of PD, highlighting the importance of universally accepted diagnostic criteria alongside personalized management approaches. Future research is needed to further expand our understanding of PD, particularly in underrepresented populations.

## Figures and Tables

**Table 1:** Case 1 and 2 laboratory results, side by side.

Test	Case 1		Case 2	
	Result [normal range]	Flags	Result	Flags
White blood cell count (WBC) (cells/μL)	4400 [4000–10000]	Normal	3500	Low
Hemoglobin (g/dL)	17.9 [11–16]	Elevated	8.3	Low
Platelets (cells/μL)	42,000 [150,000–450,000]	Low	95,000	Low
pH	7.4 [7.35–7.45]	Normal	7.4	Normal
pCO_2_ (mmHg)	20 [35–45]	Low	23	Low
pO_2_ (mmHg)	42 [80–100]	Low	43	Low
O_2_ saturation (%)	80% [95–100]	Low	84%	Low
HCO_3_ (mmol/L)	13 [22–26]	Low	18	Low
Prothrombin time (PT) (s)	22 [11–13.5 (PT-control time: 13.4)]	Elevated	20	Elevated
Partial thromboplastin time (PTT)	41 [adult: 30–45 newborn: up to 65]	Normal	42	Normal
INR	1.68 [< 1.2]	Elevated	1.48	Elevated
Aspartate aminotransferase (AST) (U/L)	97 [10–40]	Elevated	54	Elevated
Alanine aminotransferase (ALT) (U/L)	31 [10–40]	Normal	71	Elevated
Albumin (g/dL)	3.2 [3.5–5.2]	Low	2.6	Low
IgG (mg/dL)	2260 [639–1349]	Elevated	1774	Elevated
IgA (mg/dL)	454 [52–319]	Elevated	254	Elevated
IgE (IU/mL)	450 [< 160]	Elevated	489	Elevated

**Table 2:** Case 1 and 2 summary of symptoms and association with PD.

Parameter	Patient presentation		Flags	Association with PD
	Case 1	Case 2		
Age	17 years	1 year	—	
Symptoms	Progressive dyspnea, exercise intolerance, central cyanosis	Fever, seizure, productive coughs	—	Possible
Marfanoid habitus	Hypertelorism, broad nasal bridge	—	—	Yes
Skin lesions	Multiple skin lesions, hyperkeratotic areas	Reported multiple erythematous ulcers on the anal verge, oral mucositis, and crusted erythematous lesions on lips	—	Yes
Digit changes	Clubbing, cyanosis of the extremities	—	—	Yes
Hepatosplenomegaly	Hepatomegaly 2-3 cm below the costal margin, massive splenomegaly	Hepatomegaly 6 cm below costal margin, significant splenomegaly	—	Yes
Cardiac auscultation	Mild systolic murmur	—	—	No
Ophthalmic examination	Negative for lens dislocation	—	—	No
Sonographic findings	Enlarged liver, dilated portal veins, enlarged spleen with collateral vessels	Enlarged liver, an enlarged spleen with multiple hypoechoic lesions	—	Yes
Echocardiographic findings	Left ventricular hypertrophy, normal pulmonary pressure, normal systolic function	—	—	No
Blood culture	—	Positive	—	Possible
ABG (arterial blood gas)	pH: 7.4, pCO_2_: 20, pO_2_: 42, O_2_ saturation: 80%, HCO_3_: 13	pH: 7.4, pCO_2_: 23, pO_2_: 43, O_2_ saturation: 84%, HCO_3_: 18	pH: normal, pCO_2_: low, pO_2_: low, O_2_ saturation: low, HCO_3_: low	Possible
Coagulation tests	PT: 22, INR: 1.68	PT: 20, INR: 1.48	PT: elevated, INR: elevated	Possible
Liver function tests	AST: 97, ALT: 31, albumin: 3.2	AST: 54, ALT: 71, albumin: 2.6	AST: elevated, ALT: normal/elevated, albumin: low	Yes
Pulmonary perfusion scan	Escape of macroaggregated albumin from the pulmonary vasculature	—	—	Possible
Pulmonary angiography	Extensive AVMs in pulmonary vasculature	—	—	Possible?
Bone marrow aspiration and biopsy	—	Diluted bone marrow, erythroid hyperplasia, hemophagocytic cells	—	Possible
Immunoglobulin levels	IgG: 2260, IgA: 454, IgE: 450	IgG: 1774, IgA: 254, IgE: 489	IgG: elevated, IgA: elevated, IgE: elevated	Yes
Past medical history	Hepatomegaly at age 3, neck abscess	Hepatosplenomegaly at age 7 months	—	Yes

## Data Availability

Raw data files can be accessed on request.
